# Dark personality traits and entrepreneurial intentions among Pakistani university students: The role of executive functions and academic intent to entrepreneurship

**DOI:** 10.3389/fpsyg.2022.989775

**Published:** 2022-10-19

**Authors:** Rabia Khawar, Rizwana Amin, Asia Zulfqar, Samavia Hussain, Bashir Hussain, Faiqa Muqaddas

**Affiliations:** ^1^Department of Applied Psychology, Government College University, Faisalabad, Pakistan; ^2^Department of Professional Psychology, Bahria University, Islamabad, Pakistan; ^3^Department of Education, Bahauddin Zakariya University, Multan, Pakistan

**Keywords:** entrepreneurial intentions, executive functions, dark personality traits, academic intent to entrepreneurship, mediation analysis

## Abstract

This study examined the mediating role of core Executive Functions (EF: working memory and inhibitory control) and moderating role of Perceived Academic Intent to Entrepreneurship (PAIE) in relationship between Dark Personality Traits (SDT) and Entrepreneurial Intentions (EI) of university students. A sample of 539 university students enrolled in various undergraduate and postgraduate programs completed the Short Dark Triad-3 (SD3), Adult Executive Functioning Inventory (ADEXI), and measures for assessing Entrepreneurial Intent and Perceived Academic Intent to Entrepreneurship. The results showed that of SDT, only Machiavellianism was significantly associated with EI. Both of the core executive functions and PAIE were also positively correlated with EI. Moreover, findings showed that EF positively mediated the relationship between Machiavellian disposition and entrepreneurial intention, while perceived academic intent to entrepreneurship moderated the relationship between executive functions and EI. A significant moderated mediation index was also reported. Findings offer useful insight to the interplay among above mentioned variables and guide educational and organizational psychologists to employ core cognitive strategies for promoting entrepreneurial thoughts and channelizing the productive energies of students with malevolent tendencies through academic coaching.

## Introduction

Personality traits have been directly or indirectly connected to individuals’ tendencies to involve in innovative business ventures (Fayolle et al., 2014; Cardon and Kirk, 2015; Krueger, 2017). Despite the growing literature on dark triad traits within entrepreneurial context (Klotz and Neubaum, 2016; Wu et al., 2019a), yet it is difficult to derive consistent conclusions. It is therefore important to investigate the underlying mechanism of this relationship by studying various factors that possibly mediate or moderate the link between negative personality manifestations and entrepreneurial intentions related to entrepreneurship (Hoang et al., 2022).

The entrepreneurial process actually requires planning and behavior (Ajzen, 1991), which is influenced by individuals’ personality traits (Tucker et al., 2016; Farrukh et al., 2018) either positive (Brännback and Carsrud, 2018; Naz et al., 2020) or negative (Treffers, 2017). Farrukh et al. (2017) identified that personality traits not only impacts on intentions but also determined behavior followed by intentions. Dark triad mainly includes Machiavellianism, narcissism, and psychopathy as set of malevolent dispositions characterized by a tendency of self-promotion, devious nature, emotional coldness, sense of dominance, and antagonistic propensities (Paulhus and Williams, 2002; Paulhus and Jones, 2015). Besides adverse psychosocial impacts of core dark personality traits, studies have also shown some growth-oriented outcomes (Judge et al., 2009). Researchers have also elucidated the imminent benefits of the dark triad (Brunell et al., 2008; Jones and Figueredo, 2013), including its convincing role in entrepreneurial intentions across cultures (Klotz and Neubaum, 2016; Do and Dadvari, 2017).

To understand the productive aspect of a negative personality within an entrepreneurial context, it is important to unravel the thought process and cognitive attributes that account for the process of new venture creation. Cognitive processes are likely to guide the entrepreneurial intention as it involves deliberate actions (Gollwitzer and Bayer, 1999; Smith and Kosslyn, 2013). Higher order cognitive processes include human executive functioning skills (EFS) that are characterized by working memory, inhibitory control, cognitive flexibility, planning, and reasoning (Cristofori et al., 2019). These functions also enable an individual to adapt novel situations, achieve goals, and manage social interactions. Both emotional and analytical cognitive styles may influence human behavior, especially decision-making in every walk of life including entrepreneurial tendencies, intentions, decisions, and activities (Barbosa et al., 2007; Kickul et al., 2009; Krueger and Welpe, 2014). Studying particular executive functions may also help in understanding the particular cognitive styles in relation to the entrepreneurial process. So far, researchers have mainly focused on the cognitive flexibility aspect of executive functioning within an entrepreneurial framework (Dheer and Lenartowicz, 2019; Jiatong et al., 2021), while the role of working memory and inhibitory control is yet to be explored. Pihie et al. (2013) have delineated the cognitive underpinning of entrepreneurial intentions among university students from a knowledge perspective. Knowing your own thought process about a certain thing is a metacognitive ability (Schraw and Dennison, 1994) that could facilitate entrepreneurial learning.

Universities play an important role in a country’s economic growth and development by equipping its students with life-long skills and knowledge (Oketch et al., 2014). In today’s world, universities are not only considered the hub of creating, producing, and disseminating knowledge but also encourage industry engagement in terms of practicing their innovative ideas and commercialization (Striukova and Rayna, 2015) which ultimately contributes in knowledge based economy (Brown, 2016; Parmentola and Ferretti, 2018; Klofsten et al., 2019; Halbinger, 2020). It is important to seek enablers that may foster entrepreneurial intentions among university students. Entrepreneurial activities with educational set up have given rise to the concept of academic entrepreneurship that has been studied on a lesser extent (Rothaermel et al., 2007; Djokovic and Souitaris, 2008). Generally, activities at the university level involving innovation and commercialization are considered as academic entrepreneurship (Abreu and Grinevich, 2013; Wang et al., 2020). Zhang et al. (2022) accentuated the noticeable role of academic entrepreneurship in devising research activities that may generate revenue for the universities and focused on exploring the willingness of academicians toward entrepreneurial activities.

Being a middle income developing country, and despite the growing need for innovative startups due to discouraging job market trends, increasing inflation, and floating economy, Pakistan has considerably lower rates of entrepreneurial activities than others and ranked 122 out of 134 countries (Acs et al., 2017; Faghih et al., 2019). Following the guidelines provided by the Higher Education Commission, higher education institutes are now introducing entrepreneur education and developing business incubation programs to promote student startups (Higher Education Commission, 2020). It is important to develop insight among students for fruitful outcomes of these efforts. Lack of knowledge and understanding of the subject even after earning a degree in it could be the main reason for their failure in the job market (Farooq et al., 2014). The same factor may account for students’ lack of interest in entrepreneurial activities. The understanding of academic entrepreneurship is still nascent and suggests investigating the perspectives of stakeholders including university administration, teachers, researchers, and the students. Moreover, both individual and contextual factors may influence academic activities related to entrepreneurship (Perkmann et al., 2013). While advocating the extended model of planned behavior theory, Lihua (2021) highlighted the importance of student life as a foundation stage of entrepreneurial intentions and attitude. He further discussed the role of probable personal and situational factors, having substantial influence on the development of students’ entrepreneurial intention. Students’ perception and knowledge of entrepreneurial processes has been considered a good predictor of their current and later engagement in entrepreneurial activities (Pihie et al., 2013). Owing to the complexity in conceptualizing the term academic entrepreneurship in research, we prefer studying its foundation stage. For the present study, university students’ understanding of their curricula utility for new and innovative business ventures can be conceptualized as the perceived academic intent to entrepreneurship. This viewpoint gives rise to the question as to how students’ perceived academic intent to entrepreneurship may guide their entrepreneurial intentions, when combined with individual level factors including personality traits and core cognitive abilities. Considering the above mentioned gaps the present study is an effort to understand the relationship between dark triad dispositions, core executive functions, entrepreneurial intent, and perceived academic intent to entrepreneurship among university students in Pakistan.

## Literature review and hypothesis formulation

### Dark personality traits and entrepreneurial intentions

Individuals with dark personality traits violate social norms making oneself more prone to risk-taking. Machiavellianism is a personality trait that is self-serving, dishonest, strategic, and manipulative (Zettler et al., 2011; Al Aïn et al., 2013). People with narcissistic tendencies have a strong ambition to achieve personal objectives, eager to develop, and crave attention (O'Boyle et al., 2012). Psychopathy basically refers to the inability to notice, comprehend, or address emotions because of a lack of emotional intelligence and empathy. Its key characteristics include manipulation, dishonesty, ruthlessness, and a desire for intense thrill and stimulation (Akhtar et al., 2013; Crysel et al., 2013). However, due to pursuing the maximizing of their own interests and have a strong desire to dominate others (Zheng et al., 2017) these malicious dispositions especially Machiavellianism and narcissism can be helpful in planning and enacting firsthand business startups (Klotz and Neubaum, 2016).

Existing work on role of personality attributes in entrepreneurial intention mostly employs notion of Ajzen (1991), which describes entrepreneurship as a planned behavior of the related intention; and defines entrepreneurial intention as conceptual demonstrations of an individual’s tendency to launch a business (Obschonka et al., 2015; Gorgievski et al., 2018). Prior research related to negative personality traits has been investigating the dark triad of personality and explored its impact on entrepreneurial intentions (Tucker et al., 2016). Foster et al. (2011) recognized that people who indulge in self-love and seek admiration (such as narcissists) from others are more likely to be an entrepreneur as they seem to be adventurous and do not hesitate to take any kind of risk. Narcissism has been considered a favorable trait in leadership (O’Reilly et al., 2014). By setting up a new business; an individual’s psychological needs of admiration are being fulfilled which leads them to seek the administrative post (Campbell and Campbell, 2009). It is evident through literature that psychopaths have fearless nature that may lead to setting up a new business (Dutton, 2012; Morgan and Sisak, 2016). Due to their nature, such people are likely to be smart, charming (Jonason and Krause, 2013), and tend to motivate EI among students.

Moreover, researchers (Lasko and Chester, 2020; Welsh and Lenzenweger, 2021) have discussed the new term “successful psychopath,” which refers to individuals with psychopath traits responsible and tends to be leaders—entrepreneurs. People who have Machiavellianistic traits tend to be manipulative and aspire to control others for achieving goals (Sherman et al., 2013). Along with controlling others, Machiavellians seem to be persuasive and capable of making adaptive decisions even in stressful situations. Development of adaptive decisions is common in entrepreneurs. Wu et al. (2019b) and Aleksandrovna (2021) have identified that narcissism and psychopathy have a negative effect on EI whereas Machiavellianism has a positive effect. Taking the variation across existing research findings into account, we assume the following:

*H1*: Dark Triad (Machiavellianism, Narcissism and Psychopathy) will be positively correlated with entrepreneurial intentions.

### Executive functioning and entrepreneurial intentions

An individual’s desire to create a business opportunity for oneself is a cognitive state itself (Bullough et al., 2014). More specifically, researchers have found strong relationship between cognitive flexibility and different aspects of entrepreneurship, i.e., entrepreneurial competence (Kyndt and Baert, 2015; Alves and Yang, 2022), intentions (Neneh, 2019), and behavior (Simon et al., 2000; Mathews, 2017). Cognitive flexibility is an essential element of executive functioning (Miller et al., 2012). Jiatong et al. (2021) concluded that cognitive flexibility is not only positively related to entrepreneurial intentions as well as alertness but also has an indirect relation of alertness with cognitive flexibility *via* entrepreneurial efficacy. Clements et al. (2021) provided a framework that helps to understand entrepreneurial cognitions. The framework deals with how novel actions are rendered and how uncertainty is mediated by context, emotion, social cognitions, and metacognition.

Intention and behavioral control have also been identified as basic components of cognitive models of entrepreneurship (Ajzen, 1988; Karimi et al., 2012, 2013), and numerous researchers have confirmed the relationship between cognitive function with entrepreneurship intentions (Sánchez et al., 2011). Many of these researches have incorporated social cognitive perspective (Bandura, 1986) in their studies by evaluating the role of self-efficacy, perceived behavior control in entrepreneurial intentions and activities. However, the underlying mechanism of these variables and the core cognitive processes such as executive functioning skill has not been explicitly explored in relation to individual predispositions and entrepreneurial tendencies. Inhibition and working memory may also have major impact on Academic Entrepreneurial Intentions Students’ lack of business skills and entrepreneurial drive could be attributed to their lack of interest, shortage of resources, and poor understanding due to limited capacity of information processing (Almeida, 2017).

Neurocognitive underpinnings of innovative behavior may also be used to explain the entrepreneurial cognition, which will help in opportunity discovery (Beugré, 2017). Zhao and Xie (2020) have emphasized the need for considering the sources of cognitive mechanisms to explain entrepreneurial intentions. Both working memory and inhibitory control are the core executive functions that are frequently associated with top down processing, required in goal-oriented behavior and selective attention, respectively, (Diamond, 2013). These skills therefore may foster the entrepreneurial intent and also help the students to better relate their subject knowledge for entrepreneurial purposes. This leads us to propose the following hypothesis:

*H2*: Executive functions (working memory and inhibitory control) are positively correlated with entrepreneurial intentions and academic intent to entrepreneurship.

### Academic intent to entrepreneurship and entrepreneurial intentions

Entrepreneurial intentions have been introduced as an outcome variable of interest in a university context (Prodan and Drnovsek, 2010; Goethner et al., 2012; Mosey et al., 2012; Obschonka et al., 2012). However, the scarce prior research that has analyzed determinants of entrepreneurial intentions in academia has only concentrated on the individual level. Given the relevance of understanding how contextual factors can either trigger or restrain entrepreneurial intentions, both from a research and policy perspective (Lee et al., 2011; Dohse and Walter, 2012) this study simultaneously accounts for drivers of students’ inclination to engage in commercialization activities. With regard to academics and entrepreneurial intentions, studies have mostly investigated the issues of patenting, authorizing, consulting, or contract research and spin-off projects. Here again, we highlight the importance of earlier phases in the entrepreneurial process and intend to study the role of perceived academic entrepreneurial intent in university students’ entrepreneurial intentions.

Research indicates that curricula being offered at universities during degree programs enable students to develop interest in starting new business (Franke and Lüthje, 2004). Entrepreneurial education has been offered in developed countries since the 1930s (Alberti et al., 2004; Kirby and Ibrahim, 2011). However, such programs have been incorporated at each level of education-school to university (Kyrö, 2018). Whereas developing countries are facing brain drain resulting in the shortage of skilled individuals in terms of entrepreneurship (Stark, 2004). Amos et al. (2015) and Anjum et al. (2018a) have identified that entrepreneurship education (EE) is linked with entrepreneurial intention. The selection of specialized fields also related to EI, like students who opt for business courses know more about the entrepreneurial process than other subjects (Dao et al., 2021). EE enables students to learn related knowledge and develop necessary skills through innovative activities such as developing a business plan and implementing it through small-scale business (Segal et al., 2005). This practical exposure within academic settings leads to entrepreneurial attitude and intentions among students. However, not all the subjects taught at university level include entrepreneurship as a course. Moreover, most of the research on entrepreneurial intent in Pakistan is conducted on students enrolled in programs of business and management sciences (Tanveer et al., 2013; Hussain and Malik, 2018). Theory of self-concept describes that ambitions and schemas of an individual could depend on the self-assessment of the circumstances and the environment (Jakobwitz and Egan, 2006). Hence, entrepreneurial intentions can be linked with the self-assessment of students; evaluation of obtained academic knowledge to formulate strategies required for taking practical steps to execute a novel business project. This landed us to define the following hypothesis:

*H3*: Perceived academic intent to entrepreneurship will be positively correlated with university students’ entrepreneurial intentions.

### Interacted effect of EF and of PAIE on EI

To develop entrepreneurial intentions and attitudes in youth, Macquaire innovation learning and knowledge framework (Burshtein and Brodie, 2006) focuses on the categorization of the basic unit of knowledge and skills that should be part of an entrepreneurship education program. Working memory and inhibitory control as core executive functions are thought to be crucial for students’ learning capacity and knowledge inferences. Working memory serves as a workbench for information before transforming it to long-term recollections (Bergman Nutley and Söderqvist, 2017). Inhibitory control directly accentuates task-relevant knowledge, problem solving, and decision making processes. It can be concluded that the human cognitive structures comprise goal-driven control mechanisms to synchronize the level of stimulation of specific knowledge fragments and make diverting or unsolicited material in memory less reachable (Bajo et al., 2021). Students’ executive function skills therefore may interact with their understanding of the academic content for entrepreneurial purposes and both are likely to have a combined impact on entrepreneurial intentions. The following two hypotheses are put forwarded to study these assumptions:

*H4*: Perceived academic intent to entrepreneurship moderates the relationship between Executive functions and entrepreneurial intentions, i.e., the greater they perceived academic intent to entrepreneurship, the stronger the relationship between executive functions and entrepreneurial intentions.

### EF as mediator between dark triad and EI

Dark Triad is known for its egocentric and self-benefitting roles (Jones and Paulhus, 2009). Machiavellian tendencies may assist in learning both adaptive and non-adaptive strategies to survive in challenging situations (Kaplan and Gangestad, 2005). Despite the evidence for traditional deficit model (Jonason and Tost, 2010), a growing body of literature has supported the productive and adaptive impression of Machevallianism (Bereczkei and Birkas, 2014), and neurobiological studies have also glided the idea of “Machiavellian Intelligence” (Bereczkei, 2018) especially that of fluid intelligence (Kowalski et al., 2018). Although the link between inhibition and Machiavellianism is not directly confirmed; however, some researchers have suggested the connection of certain EF skills such as McIlwain (2003) reported that Machiavellians are adept to inhibit an unprompted emotive response in support of other suitable but self-oriented reaction during social interaction.

Similarly, narcissism has been regarded as having productive and adaptive features in organizational context (Ding and Hou, 2017; Al-Ghazali and Afsar, 2021). Greater confidence rather than overconfidence may influence the rational appraisal of risk taking, thus making them favorable for creating business opportunities (Foster et al., 2009). Among Dark triad, psychopathy has been negatively linked to executive function skills (Newman and Baskin-Sommers, 2011) however, recent studies have demonstrated that people with psychopathic propensities may not be related to executive function deficits, or may even show has an edge over average population (Endres et al., 2011). Despite being darker in the triad, psychopathic dispositions have been found related to successful financial outcomes such as entrepreneurial intentions explained through mechanisms of disinhibition (Walker et al., 2020). It is important to note that although the direct links between dark triad, core executive functions, and entrepreneurial intentions have not been sufficiently established and documented so far, some indirect pathways provide evidence for the potential investigation in this context. Inhibitory control is associated with problem solving skills among university students (Galarza et al., 2020) while there is evidence for the direct role of cognitive processes in explaining locus of control (Wolinsky et al., 2010). Mathieu and St-Jean (2013) found a positive relationship between internal locus of control, self-efficacy and risk taking tendencies among student entrepreneurs and also associated these variables with narcissism among them. It is therefore interesting to explore the units of executive functions for their prospective thoughtful role in entrepreneurial intentions while considering individuals’ dark dispositions simultaneously. Finally, we decided to formulate the two hypotheses:

*H5*: Machiavellianism and Narcissism will have a positive relationship with executive functions while psychopathy will be negatively associated with executive functions.

*H6*: Executive functions will mediate the relationship between Dark Triad Traits and the Entrepreneurial Intentions.

## Research methods

### Participants and procedure

This study aimed to investigate the relationship between dark triad traits, core executive functions, entrepreneurial intent, and perceived academic intent to entrepreneurship among university students in Pakistan. The study follows a correlational cross-sectional research design. Regular students of university enrolled in their final semester of undergraduate and postgraduate degree programs (BS and MS/M.Phil) were included in the study. Students enrolled in weekend programs were excluded. Students with any physical and psychological disabilities were also excluded. In Pakistan, most of the data on entrepreneurial intentions have so far been collected from students studying business, management sciences, public administration, economics, or other relevant fields as major (Anjum et al., 2019; Zreen et al., 2019; Cai et al., 2021). The students could be already familiar with the item contents; therefore, we have not included the faculty of management sciences. There are only few studies conducted with students from other disciplines (Mahmood et al., 2012; Alam et al., 2019; Ali et al., 2019). The sample of the present study was conveniently drawn (based on availability and consent) from different departments from the faculties of engineering, life sciences, basic/physical sciences, and pharmaceutical sciences of three public sectors universities of Faisalabad, Multan, and Islamabad, respectively, during a period of 4 months from February 2022 to May 2022. Considering the entrepreneurial opportunities in the market for different subjects, similarity of the programs offered at three universities and consent of the participants, the data were collected from the department of electrical engineering (29.3%), computer science and information technology (35.1%), bioinformatics/biotechnology (10.6%), food science and technology (18.7%), and the pharmacology (6.3%). A total of 580 consent forms were distributed to the students in their respective classrooms after obtaining permission from the departments. Eighteen students did not provide the consent, so the survey research forms were completed by 562 students. Data of six students were excluded as they did not meet the inclusion criteria (had some physical or mental health issue as mentioned in demographic sheet) or had incomplete forms. Data of 553 students were entered to SPSS for analysis. Furthermore, 14 cases were excluded due to missing values and outliers. Thus, the final analysis was carried out on a sample of 539 students including 55.3% men and 44.7% women (*M_age_* = 22.08; *SD_age_* = 2.15).

The study was approved by the Ethics Review Committee of the first author’s university. Data were collected from students in their classrooms after scheduling a meeting with them. Participants signed an informed consent form and were made sure that their personal information will be kept confidential. Brief instructions were given about questionnaires to the participants. Participants completed the questionnaires in one session of about 15–20 min. Lastly, we acknowledged the participants’ time and efforts.

### Instruments

All the instruments were self-report and the original English versions were used since the medium of instruction in universities is English and students easily comprehend the language.

### Demographic data sheet

Participants reported their characteristics on the demographic data sheet after signing the consent form. It included information about their gender, age, education, employment status, marital status, family size, and number of members earning the family, their parents’ education and occupation, monthly income of the family, any physical disability, and presence of any diagnosed mental or severe physical illness. Thus a detailed account of relevant characteristics was obtained.

### The short dark triad

The short dark triad (SD3; Jones and Paulhus (2014)) consists of 27 items assessing the three domains of the dark personality namely Machiavellianism, Narcissism, and Psychopathy; each subscale containing the nine items. Responses were made on a Likert type format with 1 indicating “*strongly disagree”* to 5 indicating a “*strongly agree.”* Original authors of the instrument have reported reliability coefficients ranging from 0.68 to 0.74. The measure has been used in several Pakistani studies and has also been translated into Urdu, yielding good psychometric properties >0.65 (Abbas et al., 2022; Irfan et al., 2022).

### Adult executive function inventory

Adult executive function inventory (ADEXI; Holst and Thorell, 2018) is one of the few brief self-report instruments available for assessing executive functioning (EF) in adults. It mainly focuses on the two EF domains, i.e., working memory and inhibition. Psychometric properties of ADEXI have been analyzed in different studies most recently and it was found to be a reliable and valid instrument for measuring the two important facets of executive functions. The studies confirmed the two factor structure with reliability coefficients greater than 0.80 (Holst and Thorell, 2018; López et al., 2021).

### Entrepreneur intentions

Entrepreneur Intentions (EI) were measured through six items, which were derived from existing measures used by Miranda et al. (2017) who originally adopted the scale developed by Liñán and Chen (2009). Responses were recorded on the five point Likert type scale ranging from 1 (strongly disagree) to 5 (strongly agree). EI is a reliable and valid measure having excellent internal consistency of 0.89 and has been frequently used by researchers in Pakistan (Anjum et al., 2018b; Sarwar et al., 2021).

### Perceived academic intent to entrepreneurship

In order to measure the academic intent to entrepreneurship, five items were developed by the authors of present study after consulting experts in the field. After a pilot test with 20 students, items were revised for further clarity. Cognitive debriefing with five more students was done to seek better interpretation of each item by the targeted population. No change was indicated. Items were later rated on a scale of 1–7 for their relevance to the construct being assessed. Ten experts from the field of psychometrics and business administration evaluated the items. Intra class coefficients for all the items were greater than 0.80 while Kappa coefficient was computed for the total score that yielded a value of 0.87. For the current study, participants rated their responses on five-point Likert type format in which 1 indicated *strongly disagree* and 5 indicated *strongly agree*. Here is the sample item from the scale, *“My degree can play an important role in starting a business endeavor.”*

### Data processing and analytic strategy

Firstly, missing values were analyzed and cases with 10% or more missing responses were removed from the dataset. In contrast, missing values <10% were replaced with the serial mean method using missing values analysis in SPSS. Outliers were identified and excluded to ensure the normal distribution of the data. Further analyses were performed on a final data set of 539 participants. We computed descriptive and inferential statistics by using the SPSS 23.0 version. Internal consistency reliability coefficients of measures were estimated using Cronbach’s Alpha (Cronbach, 1951) and Mc. Donald’s Omega coefficients (McDonald, 1999). Additionally, we conducted Confirmatory Factor Analysis (CFA) for assessing the validity of newly developed five items, a brief measure of perceived academic intent to entrepreneurship by using AMOS 23.0 version. Pearson Product Moment Correlation was used to analyze interrelationship of study variables and also for the demographic correlates. Moderated mediation analysis was computed through PROCESS MACRO (Hayes, 2022).

## Results

The study was designed to examine the relationship between dark triad traits, core executive functions, entrepreneurial intent, and perceived academic intent to entrepreneurship among university students in Pakistan. This section includes descriptive statistics for participants’ demographic characteristics, means, and SDs for the total scores and results of reliability analysis. Results of CFA for self-developed items assessing perceived academic intent to entrepreneurship are also illustrated. It further includes findings of moderated mediation.

We firstly present the demographic characteristics of the participants in Table 1. It included gender, age, type of degree, total monthly income (in PKR), family size, employment status, parents’ education, and occupation etc. The table shows means and standard deviations for continuous variables (age, monthly income, family size, and earning members in family), and frequencies for categorical variables (gender, type of degree, and parents’ education level and occupation). The majority of the students were enrolled in undergraduate programs (79.4% last semester) while the rest were almost equally distributed in postgraduate (MS/M.Phil) and professional degrees (Law and Pharm-D). Results revealed that there were on average seven members in a family where hardly two members earned for the whole family. Most of the students were not earning themselves (78%) while a few identified themselves as employed (22%). On the other hand, 30% respondents reported that they are dependent on their fathers and yet none of the students showed involvement in business activities.

**Table 1:** Socio-demographic characteristics of the study participants.

Variables	Groups	*M(SD)*	*f (%)*
Age		22.08 (2.15)	
Family size		6.66 (3.51)	
Monthly income		125806.18 (662482.32)	
Earning members in the family		1.71 (1.03)	
Gender			
	Men		298 (55.3)
	Women		241 (44.7)
	Total		539 (100)
Education			
	Undergraduate program		428 (79.4)
	Postgraduate program		58 (10.8)
	Professional Degree		53 (9.8)
	Total		539 (100)
Fathers’ education			
	Elementary level		84 (15.6)
	Secondary/higher secondary level		203 (37.7)
	Graduation level		205 (38.0)
	Post-graduation level		45 (8.3)
	Others		2 (0.4)
	Total		539 (100)
Fathers’ occupation			
	Business		161 (29.9)
	Private/public job		209 (38.8)
	Farming/agriculture		60 (11.1)
	Retired		28 (5.2)
	Unemployed		9 (1.7)
	Others		72 (13.4)
	Total		539 (100)
Mothers’ education			
	Elementary level		155 (28.8)
	Secondary/Higher secondary level		215 (39.9)
	Graduation level		147 (27.3)
	Post-graduation level		15 (2.8)
	Others		6 (1.3)
	Total		539 (100)
Mothers’ occupation			
	Working		83 (15.4)
	Non-working		449 (83.3)
	Others		7 (1.3)
	Total		539 (100)
Employment status			
	Employed		120 (22.3)
	Not employed		419 (77.7)
	Total		539 (100)

### Reliability analyses and descriptive statistics

Table 2 demonstrates descriptive statistics and reliability coefficients for the scales used in the present study. Measures of Entrepreneurial Intent and Perceived Academic Intent to Entrepreneurship showed good alpha and omega reliability coefficients (> 0.75; Tavakol and Dennick, 2011; Green and Yang, 2015). Reliability estimates for Adult Executive Functioning Inventory were also adequate (> 0.70; Tavakol and Dennick, 2011; Green and Yang, 2015). The Short Dark Triad yielded somewhat lower reliability coefficients (*α* ***=*** 0.63, ω **=** 0.64), however these could be considered acceptable (Green and Yang, 2015; Ursachi et al., 2015). Moreover, the reliability coefficients of SD3 are comparable to the internal consistency reported by the original authors (Jones and Paulhus, 2014). Both skewness and kurtosis values ranged between ±0.5 depicting normal data distribution (Meyer et al., 2015).

**Table 2:** Descriptive statistics and reliability estimates for the study measures.

Scales					Skewness		Kurtosis	
	*K*	*M(SD)*	*α*	ω	Statistics	*SE*	Statistics	*SE*
SD3 Total	27	8.72(−1.14)	0.63	0.64	0.090	0.105	−0.204	0.210
ADEXI	14	6.12(1.19)	0.73	0.73	−0.112	0.105	−0.006	0.210
EI	6	3.64 (0.92)	0.86	0.86	−0.456	0.105	−0.390	0.210
PAIE	5	3.63 (0.89)	0.79	0.80	−0.495	0.105	−0.168	0.210

SD3, Short Dark Triad; ADEXI, Adult Executive Function Inventory; EI, Entrepreneur Intentions; and PAIE, Perceived Academic Intent to Entrepreneurship.

### Confirmatory factor analysis

**Figure 1:**
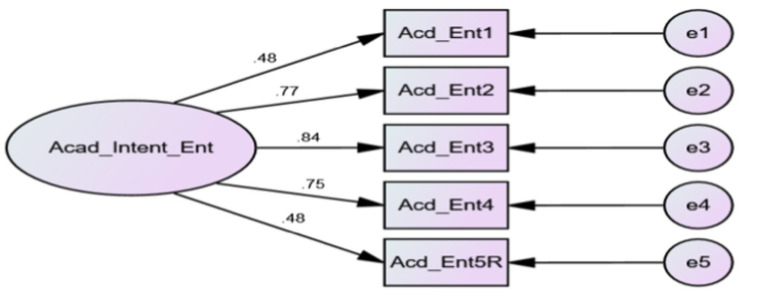
Confirmatory factor analysis (CFA) for brief measure of perceived academic intent to entrepreneurship.

Confirmatory factor analysis (CFA) was performed using AMOS (Version 23) for Perceived Academic Intent to Entrepreneurship Scale (see, Figure 1). Findings of fit indices of CFA model are presented in Table 3 presenting the model as a perfect fit (Marsh et al., 2020).

**Table 3:** Factor loadings for perceived academic intent to entrepreneurship (PAEI scale).

Items	Factor loadings
1.	0.48
2.	0.77
3.	0.84
4.	0.75
5.	0.48
CR	0.804

Factor loadings of Perceived Academic Intent to Entrepreneurship Scale and composite reliability were shown in Table 4. Factor Loadings for the PAIE ranged from 0.48 to 0.84; while the composite reliability was good (0.80). A loading value of 0.70 or higher is considered as general recommendation for ensuring better reliability of the items since the measured construct could explain 50% of the variance in the indicator used for analysis (Hair et al., 2019). In current study we set a fair item loading (>0.45) criteria for factor analyses as recommended by Tabachnick and Fidell (2007). Removal of items does not solely depend on the loadings and following the recommendation of removing the items with loading values below 0.40 (Hair et al., 2011, 2017), we retained item numbers 1 and 5 despite having relatively low factor loadings (0.48). Several researchers have used this criterion to avoid unnecessary exclusion of items without further exploration with diverse samples from the same population (especially when the newly developed scale is used for the first time); (Bardi et al., 2021). Reversed scoring item (5) could also be a reason for this poorer loading (…).

**Table 4:** CFA model fit indices for perceived academic intent to entrepreneurship (PAIE scale).

Fit indices	Current model values	Acceptable fit values	Good fit level values
χ^2^/df	2.3	≤ 4.0–5.0 (Wheaton et al., 1977)	≤ 3.0 Tabachnick and Fidell, 2007
CFI	0.99	≥ 0.90 (Browne and Cudeck, 1993)	≥ 0.97 (Hu and Bentler, 1999)
TLI	0.98	≥ 0.90 (Bentler and Bonett, 1980)	≥ 0.95 (Schumacker and Lomax, 2004)
AGFI	0.97	≥ 0.85 (Hooper et al., 2008)	≥ 0.90 (Anderson and Gerbing, 1988)
GFI	0.99	≥ 0.85 (Yılmaz and ve Çelik, 2009)	≥ 0.90 (Anderson and Gerbing, 1988)
IFI	0.99	≥ 0.90 (Bollen, 1989)	≥ 0.95 (Tabachnick and Fidell, 2007)
NFI	0.98	≥ 0.90 (Byrne, 2001)	≥ 0.95 (Schumacker and Lomax, 2004)
RMSEA	0.05	≤ 0.06–0.08 (Meydan et al., 2011)	≤ 0.05 (Browne and Cudeck, 1993)

### Common method bias

Harman’s single-factor analysis was executed to test common method bias in data. This method proposed by Harman (1976) that examines whether variations in the data are accounted for by only one variable. If a single variable accounts for more than 50% of the variance in the data, then the common method bias is present (Podsakoff et al., 2003). Results from the rotated factor matrix show seven extracted items, with the first factor having 10.37% of the total variance explained. Thus, there was no possible issue of common method bias in the data of present study.

### Correlation analysis

Results of inter-correlation among study variables were presented in Table 5. Machiavellianism was positively correlated with entrepreneur intentions (*r* = 0.10, *p* < 0.05), perceived academic intent to entrepreneurship (*r* = 0.16, *p* < 0.001), working memory (*r* = 0.13, *p* < 0.01), inhibition (*r* = 0.10, *p* < 0.05) and also the overall scores on adult executive functioning inventory. Narcissism was only significantly associated with perceived academic intent to entrepreneurship (*r* = 0.10, *p* < 0.05). It yielded no significant relationship with entrepreneurial intent or any of the executive functions. A significant positive correlation was found between psychopathy and executive functions, both working memory (*r* = 0.19, *p* < 0.001), and inhibition (*r* = 0.27, *p* < 0.001). Working memory (*r* = 0.13, *p* < 0.01) and inhibition (*r* = 0.11, *p* < 0.05) were directly significantly linked with entrepreneur intentions, respectively. There was a strong positive relationship between perceived academic intent to entrepreneurship and the entrepreneur intentions. However, perceived academic intent to entrepreneurship did not significantly correlate with both of the executive functions.

**Table 5:** Inter-correlation among demographics and study variables (Dart personality traits, Executive functioning (Working memory, Inhibition), Entrepreneur intentions, and Perceived academic intent to entrepreneurship).

Variables	1	2	3	4	5	6	7	8	9	10	11	12	13
1.Age	1												
2. Gender	−0.14^**^	1											
3. Family Size	−0.08	−0.07	1										
4. Education	0.39^***^	0.004	−0.04	1									
5. Employment Status	−0.23^***^	0.15^**^	0.10^*^	−0.06	1								
6. Machiavellianism	0.01	−0.02	0.06	0.09^*^	−0.05	1							
7. Narcissism	0.06	−0.05	0.02	0.03	−0.03	0.23^***^	1						
8. Psychopathy	0.04	−0.14^**^	0.10^*^	0.01	−0.10^*^	0.22^***^	0.18^***^	1					
9. Working Memory	−0.10^*^	−0.04	0.07	−0.12^**^	−0.04	0.13^**^	−0.03	0.19^***^	1				
10. Inhibition	−0.07	−0.01	0.02	−0.07	−0.01	0.10^*^	0.03	0.27^***^	0.46^***^	1			
11. Executive Functions Total	−0.10^*^	−0.03	0.05	−0.11^*^	−0.03	0.13^**^	0.002	0.27^***^	0.84^***^	0.87^***^	1		
12. Entrepreneur Intentions	0.01	−0.18^**^	−0.02	−0.02	0.02	0.10^*^	0.08	0.04	0.13^**^	0.11^*^	0.14^**^	1	
13. PAIE	0.03	−0.02	−0.05	0.07	0.05	0.16^***^	0.10^*^	−0.08	0.003	−0.01	−0.01	0.51^***^	1

PAIE, Perceived Academic Intent to Entrepreneurship.

*** *p* < 0.001;

** *p* < 0.01;

* *p* < 0.05.

Apart from these findings the inter-correlation among demographics and study variables were also reported and only a few emerged as significant correlates. Age showed a significant negative relationship with working memory (*r* = −0.10, *p* < 0.05). Students from larger families showed greater psychopathic tendencies (*r* = 0.10, *p* < 0.05). Having greater family size was positively linked to psychopathic tendencies (*r* = 0.10, *p* < 0.05) while employment status was inversely associated with psychopathy (*r* = −0.10, *p* < 0.05). Students who were employed showed lesser psychopathic tendencies. Education was significantly positively associated with Machiavellianism (*r* = 0.09, *p* < 0.05) and showed a significant inverse relationship with working memory (*r* = −0.12, *p* < 0.05). It indicated that students of higher level of education (post graduate and professional degree programs) scored higher on Machiavellianism and lower on working memory. However, the strength of these relationships was adequate only.

### Moderated mediation analysis

**Figure 2:**
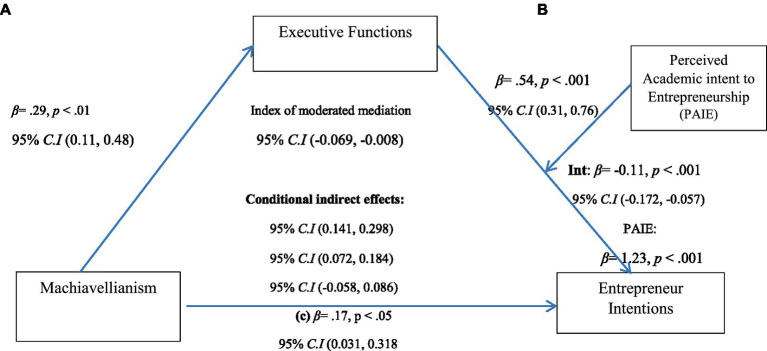
Moderated mediation measurement model.

We executed parametric bootstrapping analyses with 5,000 bootstraps (with significance level determined at 95%; Preacher et al., 2007) for testing moderated mediation using model 14 of PROCESS macro (Hayes, 2022), assuming Perceived Academic Intent to Entrepreneurship (PAIE) as a moderator in a mediated path (b) from Machiavellianism to Entrepreneur Intentions (EI) through executive functions (EF; Table 6). Results showed that Machiavellianism initially significantly predicted entrepreneurial intentions (*β* = 0.18, *p* < 0.01; 95% C. I = 0.031, 0.318) and the executive functions (*β* = 0.29, *p* < 0.01; 95% C. I = 0.110, 0.480). Furthermore, direct effect of IV (Machiavellianism) on DV (Entrepreneur intentions) was insignificant (*β = 0*.001*, p = ns, t = 0*.008) while executive functions significantly contributed to entrepreneur intentions (*β* = 0.54, *p* < 0.001; 95% C. I = 0.315, 0.763). Findings suggested that executive function was a significant mediator between the relationship of Machiavellianism and entrepreneurial intent (Figure 2). Findings of mediated moderation analysis revealed that perceived academic intent to entrepreneurship significantly moderated the relationship between Executive Functions and Entrepreneur Intentions (*β* = 1.24, *p* < 0.001; 95% C. I = 0.869, 1.592) accounting 30% of variance. Conditional indirect effects of moderator are presented in Table 7 showing that on low and medium levels of perceived academic intent, the relationship between EF and EI was positive and significant, whereas it was insignificant on the higher end of PAIE. Significant moderated mediation index was shown in Table 8.

**Table 6:** Models summary for Path A and Path B.

					*CI 95%*		
Variables	*B*	*SE*	*t*	*p*	*LL*	*UL*	*R* ^*2*^
**Path A (IV ➔ M)**							
Machiavellianism	0.29	0.095	3.10	0.002	0.108	0.480	0.02
**Path B**							
Machiavellianism	0.001	0.063	0.008	0.993	−0.123	0.124	0.30
Executive Functions	0.54	0.114	4.72	0.000	0.315	0.763	
Academic Intent	1.23	0.184	6.69	0.000	0.869	1.592	
Int_1 *R^2^* Change = 0.020	−0.114	0.029	−3.91	0.000	−0.172	−0.057	

**Table 7:** Conditional indirect effects of X on Y.

PAIE	Effect	Boot SE	Boot LLCI	Boot ULCI
2.80	0.067	0.027	0.020	0.128
3.63	0.037	0.015	0.011	0.070
4.53	0.006	0.010	−0.015	0.027

C Prime Path IV ➔ M ➔ DV; PAIE, Perceived Academic Intent to Entrepreneurship. Machiavellianism ➔ Executive Functions ➔ Entrepreneur Intentions.

**Table 8:** Index of moderated mediation analysis.

	Index	Boot SE	Boot LLCI	Boot ULCI
**Academic Intent**	−0.034	0.016	−0.069	−0.007

## Discussion

The present study was designed to investigate the relationship between dark triad traits, core executive functions, entrepreneurial intent and perceived academic intent to entrepreneurship among university students in Pakistan. The study evaluated the mediating role of core executive functions in relationship between dark dispositions and entrepreneurial intent of university students. Moreover, perceived academic intent to entrepreneurship was also investigated for its impact on entrepreneurial intentions through executive functions and dark personality traits. The findings partially supported the primary assumptions about interrelationship among study variables. Only one of the three dark personality traits, Machiavellianism, was significantly associated with entrepreneurial intentions. However, the strength of the relationship was not promising as compared to the results from existing studies which have shown a strong direct relationship between Machiavellianism and innovative business start-up intentions (Wu et al., 2019a). People with Machiavellian tendencies are known for being more focused about their interests and are also reported as self-motivated and therefore, are more likely to engage in entrepreneurial planning and activities (Cai et al., 2021). Machiavellian orientation also stands out among the dark traits because of its non-clinical nature and more relevance to the organizational and social contexts (Fehr et al., 1992; Rogoza and Cieciuch, 2020).

For narcissism and psychopathy, some studies have reported a positive relationship between narcissism and entrepreneurial intent and behavior (Hmieleski and Lerner, 2016), while others have shown an inverse one (Wu et al., 2019b). Studies conducted in Pakistan have also supported this association (Li et al., 2020; Zia et al., 2020), however, in the present study, narcissism and psychopathy were found unrelated to entrepreneurial intentions. There is also evidence for the mixed findings on the direct link between negative dispositions and EI. Entrepreneurship could be more attractive for individuals with dark personality traits (Brownell et al., 2021), yet results may differ across diverse populations such as successful entrepreneurs, professionals, and students (Liang, 2018). Therefore, we suggest a careful interpretation of the findings.

Both Machiavellianism and Psychopathy showed significant positive relationships with working memory and inhibitory control aspects of executive functions. Machiavellianism was more strongly associated with working memory than its relationship with inhibitory control. Good working memory abilities are supportive of the integral manipulative skills and tactful nature of Machiavellian personality (Bereczkei and Birkas, 2014; Simon et al., 2015) and inhibitory control may help in regulating their controlled and self-oriented reaction to a certain context for obtaining a desirable outcome (McIlwain, 2003; Fatima and Shahid, 2020). Significant positive relationship between psychopathic tendencies and working memory, and more pronounced association with inhibitory control is however less documented. Though most of the literature in psychology supports the deficit hypothesis of executive function for individuals with psychopathic traits (Delfin et al., 2018; Pasion et al., 2018); different facets of psychopathy may be differently associated with various executive functions. While psychopathic features like social disaffection and disbelief are linked with working memory deficits, other facets like self-centeredness and antagonism may not be related to the impaired working memory. Individuals having impressiveness and manipulation as more dominating aspects of their psychopathic tendencies, are likely to learn impulse control (Lasko and Chester, 2020).

Moreover, the secondary spectrum of psychopathy is more externalizing in nature and has been frequently studied in relation to forensic and clinical settings (Sadeh and Verona, 2008). Findings of the current study with regard to the positive link between psychopathic traits and both working memory and inhibition are in line with the new yet controversial model of “successful psychopath” (Palmen et al., 2020). It is considerable to have diverse findings across forensic/non-forensic, clinical and non-clinical samples such as university students in the present study. Attention and mental focus are essential characteristics of EI (Lerner et al., 2019) so basic executive functions of working memory and inhibitory control were assumed to positively link with it. Findings of the current study strongly supported this notion as both working memory and inhibition were significantly associated with Entrepreneurial Intentions. Strength of relationship however, was slightly greater for working memory as compared to inhibition. Cognitive resources, such as metacognitions, cognitive flexibility, and cognitive load have been associated with EI by different researchers (Haynie et al., 2012; Liang et al., 2016; Jiatong et al., 2021).

Working memory capacity is the triggering factor behind higher mental abilities considered necessary for thoughtful actions (Engle and Kane, 2004) that are also reflected in entrepreneurial processes. Taking a top down approach into account for explaining EI, working memory follows the same line to facilitate goal-directed behavior (Pessoa and Ungerleider, 2004) and is likely to inspire the inhibition process simultaneously. Therefore, a similar mechanism may explain the link between inhibitory control and EI as inhibitory control enables the person to suppress the unwanted options while targeting the required ones for more suitable actions (Diamond, 2013). Thus, planning and enactment related to entrepreneurial intentions may be considered a result of both of the executive functions that further connects to cognitive flexibility. Unexpectedly, both of the core executive functions did not significantly correlate with perceived academic intent to entrepreneurship.

Perceived academic intent to entrepreneurship (PAIE) also yielded significant correlation coefficients with Machiavellianism and Narcissistic tendencies. Both of the dark triad traits are linked with pursuit of individual goal interests and goals and the selfish motive underlying these traits enhances their chance to benefit financially and professionally (Effelsberg et al., 2014; Castille et al., 2018). Kareshki (2011) also established the link between some facets of Machiavellian beliefs and goal-orientation particularly in academic settings. Their motivational, creative, and innovative capacities (Kapoor, 2015; Lebuda et al., 2021) may also facilitate them to perceive the academic content in a different way for its future economic utilities as compared to their fellows. Psychopathic propensities were found unrelated to PAIE.

Lastly, results of moderated mediation analysis showed that the influence of executive functioning skills on the relationship between Machiavellianism and entrepreneurial intent was moderated significantly by perceived academic intent to entrepreneurship. Even though PAIE was found unrelated to EF directly, interaction of both the variables affected the relationship between Machiavellianism and the effect was more pronounced and positively significant at the lower to moderate levels of PAIE. Overall executive functions (total score on AEFI) also significantly mediated the relationship between Machiavellianism and entrepreneurial intentions. It demonstrates that the impact of this manipulative trait is diminished for EI when taking working memory and inhibitory control into account. Present findings suggest using the instruments from the cognitive tool box for better understanding of entrepreneurial cognitions including intentions as suggested earlier by Baron and Ward (2004). An understanding of these underlying psychological processes may help developing strategies for nurturing entrepreneurial tendencies and outcomes through knowledge structures and individual modes of thinking.

## Implications and limitations of the study

The findings of current study are valuable addition to the area of entrepreneurial intentions as it uniquely combines the vastly studied aspects (personality) to sparsely documented areas (core executive functions) and also introduces the novel dimension of perceived academic intent to entrepreneurship. This study emphasized how dark personality traits and executive functioning play a role in developing Entrepreneurial Intention among university students and proposes an important implication for subject experts, educationists, educational psychologists, and other stake holders involved in curriculum development. Learning to employ knowledge for financial outcomes among students may be cultivated through interventions that enhance executive functioning skills. Moreover, students’ negative energy based on dark dispositions can be channelized for more productive outcomes. Understanding of executive functioning profiles of individuals with greater Machiavellian tendencies may help counselors and psychologists in identifying the precise role of EFs in planning and achieving the productive and realistic goals for financial outcomes. Demographic correlates also showed promising implications especially with reference to gender and employment status. Males are more inclined to engage in EI than females while lesser psychopathic tends in employed students indicate that involvement in economic activities may suppress the negativity in individuals’ personality. Therefore, we emphasize the need to focus entrepreneurial education and activities in university students especially in female students also.

The study also had some limitations. As to sample, we have only involved three universities from Islamabad, Multan, and Faisalabad taking into account the available resources; however, future researches can involve more universities to get a bigger picture of the phenomena under study. Moreover, the sample was restricted to university students studying in the last semester of graduate, post graduate, or professional degree programs. That was also the reason for the smaller sample size. Data from students enrolled in different semesters will only help in increasing the sample size but may provide useful insights on the developmental course of entrepreneurial intention during academic life. Use of longitudinal design over cross-sectional one will further enhance the understanding of this developmental course. Students were not equally distributed across faculties (life sciences, computer sciences, basic and social sciences etc.). We considered a limited number of departments while future researchers should plan comparative studies across different faculties to identify the gaps on academic aspects of entrepreneurial processes. Reliability of SD3 was somewhat low although it was comparable to ranges yielded by this scale across cultures and populations (Jones and Paulhus, 2014). Only three dark traits were assessed in the present study, while future researchers may take sadism being fourth and more recent one into account in relation to executive functions and entrepreneurial intention.

Psychometric issues can be addressed, by using indigenously developed measures as the one developed in the current research for PAIE which yielded better psychometric properties that could be improved in future studies. Academic entrepreneurship is a complex phenomenon that can cover a diverse range of topics and populations. There is still a need for more concrete operational definition of the construct and relevant assessment tools. Maximum stakeholders should be taken into account while conducting research on its aspects. Present study was limited to students; future studies may obtain data from researchers, teachers, and entrepreneurs, administrators of the institutions, patent holders, research grant winners, and PhD scholars to compare the results and establish the links. Moreover, qualitative studies should be encouraged to gain a better comprehension of the concepts related to entrepreneurship and its psychosocial correlates.

## Data availability statement

The raw data supporting the conclusions of this article will be made available by the authors, without undue reservation.

## Ethics statement

The study was approved by the Ethics Review Committee of Government College University, Faisalabad, Pakistan. Human participants’ rights to information, willingness, confidentiality, and withdrawal were regarded, and written informed consent was obtained.

## Author contributions

RK proposed the current study. RK, RA, and AZ equally contributed to designing the methodology, writing, reviewing, and finalizing the manuscript. SH and FM along with BH collected data from the field and also assisted in statistical analysis and manuscript writing. All authors contributed to the article and approved the submitted version.

## Conflict of interest

The authors declare that the research was conducted in the absence of any commercial or financial relationships that could be construed as a potential conflict of interest.

## Publisher’s note

All claims expressed in this article are solely those of the authors and do not necessarily represent those of their affiliated organizations, or those of the publisher, the editors and the reviewers. Any product that may be evaluated in this article, or claim that may be made by its manufacturer, is not guaranteed or endorsed by the publisher.
